# Systemic evolutionary chemical space exploration for drug discovery

**DOI:** 10.1186/s13321-022-00598-4

**Published:** 2022-04-01

**Authors:** Chong Lu, Shien Liu, Weihua Shi, Jun Yu, Zhou Zhou, Xiaoxiao Zhang, Xiaoli Lu, Faji Cai, Ning Xia, Yikai Wang

**Affiliations:** 1 Keen Therapeutics Co., Ltd., Shanghai, China; 2 Chemical.AI, Shanghai, China

**Keywords:** Chemical space exploration, Fragment-based drug discovery, Deep learning, De novo drug design, PHGDH

## Abstract

Chemical space exploration is a major task of the hit-finding process during the pursuit of novel chemical entities. Compared with other screening technologies, computational de novo design has become a popular approach to overcome the limitation of current chemical libraries. Here, we reported a *de novo* design platform named systemic evolutionary chemical space explorer (SECSE). The platform was conceptually inspired by fragment-based drug design, that miniaturized a “lego-building” process within the pocket of a certain target. The key to virtual hits generation was then turned into a computational search problem. To enhance search and optimization, human intelligence and deep learning were integrated. Application of SECSE against phosphoglycerate dehydrogenase (PHGDH), proved its potential in finding novel and diverse small molecules that are attractive starting points for further validation. This platform is open-sourced and the code is available at http://github.com/KeenThera/SECSE.

**Supplementary Information:**

The online version contains supplementary material available at 10.1186/s13321-022-00598-4.

## Introduction

Developing a new drug is an enduring process that is estimated to take 10–15 years with a cost of 1.5 billion US dollars or more. At the early drug discovery stage, the hit-finding program is crucial for a successful R&D campaign, especially for the challenging targets, which usually yield meager hit rates. There are many options for hit-finding, such as high-throughput screening (HTS), affinity selection-mass spectrometry (AS-MS), fragment-based drug design (FBDD), DNA-encoded library technology (DELT), and virtual screening (VS). However, all the above approaches suffer from the requirement of a predefined (real or virtual) compound library. To address the limitation, make-on-demand libraries [1–3] have gained some recent popularity in expanding the chemical space. Nevertheless, even the most extensive collection of compounds claimed so far with the size of $10^{26}$ [4, 5] is still a very tiny fraction of the estimated chemical space in the order of $10^{63}$ [6]. Therefore, a systemic chemical space searching strategy is needed to provide optimal starting points against the target of interest.

De novo design is one such strategy that is conceptually able to overcome the limitation of existing compound libraries, which produces novel compounds based on the 3D crystal structure of a given target from scratch. A comprehensive summary [7–11] of the recent development in de novo design is out of the scope of this paper though, several seminal works that inspire us will be briefly reviewed in the following section.

LUDI [12] was an example of early attempts, where fragments from a predefined library were positioned into sub-pockets of the target. Then the fitted fragments were bridged together to form a new compound that better occupied the pocket. A similar approach called LigBuilder [13] used module POCKET [14] to analyze and parameterize protein pockets and then applied module GROW or LINK to build up new molecules. A genetic algorithm was implemented in the growing and linking steps to avoid the combinatorial explosion of the molecular generating process. Subsequently, module SCORE predicted the binding affinity of the molecules. Synthesis accessibility (SA) analysis and more druglike filters were incorporated in the upgraded program LigBuilder v2 [15]. While in the latest version LigBuilder v3 [15], the authors began to consider the flexibility of pockets by including several samples from a particular target or different targets with similar binding pockets in the generation workflow. Wang et al. developed two versions of AutoT&T [16, 17] to automatically generate analogs for hit compounds under the spatial constraints of the targeted binding pocket. Their valuable attempts opened a way for computational de novo design methods in lead optimization. OpenGrowth [18] was an open-sourced de novo design program which also based on the fragment-based growing strategy. The 3-mers screening method required that generated molecules be made by defined fragments derived from the drug library, which warranted druglike properties. Like LigBuilder v3, different conformations of the target were considered to address the protein flexibility issue. Durrant *et al.* developed AutoGrow [19] to integrate fragment-based growing and docking with an evolutionary algorithm. The latest version is AutoGrow4 [20], which employed reaction-based rules for growing as mutation operators in the genetic algorithm and merging two molecules with maximum common substructure as crossover operators. Substructure or property filters (like the rule of 5 [21], PAINS [22]) were used to control the quality of generated molecules. At the same time, open-source docking programs were invoked to evaluate the binding affinity. Although AutoGrow4 performed well in some cases, reaction-based molecular generation is intrinsically limited for constructing novel chemical entities. Polishchuk published an open-sourced tool called CReM [23] to produce highly diverse structures by fragment manipulation (mutate, grow and link). Nigam *et al.* proposed STONED for efficient search of chemical space using a SELFIES modification method [24]. Recently, Steinmann and Jensen reported a non-fragment-based approach [25], which used a set of reaction-like rules to build up chemical structures, yielding molecules with acceptable glide docking scores and synthetic feasibility by genetic algorithm.

In addition to rule-based generators, deep generative models have also been extensively explored. MolAICal [26] used generative deep learning models for 2D structure construction and classical methods for 3D evaluation and simulation. Recently, Ma *et al.* [27] developed SBMolGen, which contained an RNN based SMILES generator called ChemTS [28], a Monte Carlo tree search, and docking simulations. Lai *et al.*, the authors of LigBuilder, developed DeepLigBuilder [29] to generate 3D molecules directly from deep generative models. Several other new approaches utilizing deep learning methods to generate 3D molecules have been reported [30–33]. Compared with 1D/2D generative models or rule-based methods [34, 35], the competitive advantage of using these 3D models is direct 3D conformation generation with high speed. However, it is not easy to directly converge when training deep learning models end to end. Researchers have to introduce some special treatments for the data type and model architecture to terminate the training process, which is usually difficult to interpret.

Inspired by previous attempts in the field, we present our work setting up a platform to explore the chemical space against a given target systemically. Analogous to other programs, the SECSE platform consists of three modules, namely, a molecular generator, a fitness evaluator, and a genetic selector. The output is chemical structures that specifically fit the evaluation model for a defined pocket or other criteria. Moreover, PHGDH is chosen to demonstrate the potential of the SECSE platform. Virtually generated molecules are shown, and the corresponding structure-activity relationship is analyzed for this target. Their high docking scores and reasonable binding poses, in addition to structural novelty and patentability, warrant further exploration.

## Implementation

SECSE is a de novo design software that mainly combines rule-based molecular generation and structure-based drug design methods using genetic algorithms, as well as a deep learning module. The SECSE platform is implemented by a molecular generator, a fitness evaluator, and a genetic selector. In the molecular generator module, we have created more than 3000 rules for molecular transformations based on knowledge and expertise from the literature domain and our internal medicinal chemists. These rules are comprehensively curated and strategically categorized for optimal output. In the fitness evaluator module, molecular docking is utilized for compound assessment, which can also be replaced by shape-/pharmacophore-based evaluation methods. In the genetic selector module, a genetic algorithm is used given the similarity between the triage strategy of fragment growing and the genetic rule “fitness to survive”.

**Fig. 1:**
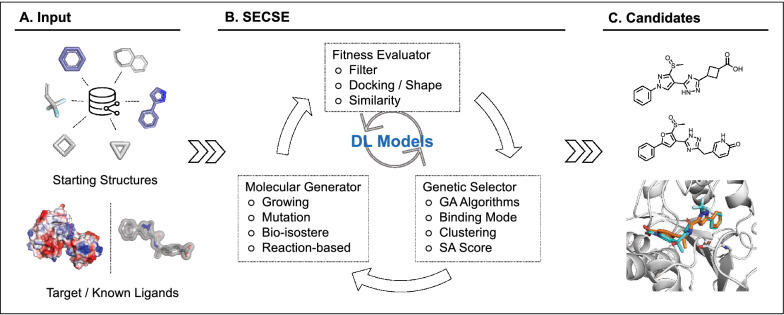
The general workflow of SECSE. **A** Fragment library or preferred structures can be used as starting point for molecule evolution. Either binding pocket of 3D protein structures (structure-based) or a set of known active ligands (ligand-based) can be used for fitness evaluation. **B** SECSE has three basic modules, molecular generator, fitness evaluator, and genetic selector. **C** Examples of generated structures and binding poses can be analyzed for virtual candidate prioritization. Protein structure is shown in white cartoon. A selected candidate is shown in cyan stick, while reference compound is shown in orange stick

The workflow of SECSE is described in Fig. 1. In the first place, fragments/groups are docked/positioned into the pocket, from which the ones with high docking scores or ligand efficiency are picked as elites. It is noteworthy to point out that fragments with less than 13 heavy atoms are exhaustively enumerated as initial input, yet any given structures or functional groups can be used as starting points. Then all the elites are evolved to generate novel chemical structures by applying the rules. The child molecules are clustered and sampled to represent the pool. The sampled molecules are docked into the pocket again. Highly scored molecules adopting hereditary or reasonable 3D orientation are chosen as new elites. This process concludes one cycle. After multiple cycles of iteration, a considerable number of compounds are generated and accumulated. To comprehensively evaluate all compounds, we introduced a graph-based machine learning module to speed up elite selection in each generation. Finally, hit compounds are visually inspected and selected before wet lab synthesis.

### Fragments collection

As starting points of the entire workflow, the quality of the fragment collection would determine the final output to some extent. Fragments derived from compounds in co-crystal structures or based on hypotheses can be used as proprietary input. However, it would inevitably be limited by the size of existing fragment collections or human bias. To ensure the diversity of the starting fragment library, we proposed an algorithm analogous to GDB13 [36] that can potentially enumerate molecules containing up to 12 heavy atoms with MW ranging from 50 Da to 210 Da. We provided the source code in order to explicitly present the details of the fragment space exploration.

As described in Additional file 1, sequential carbon strings, such as “CCCCCC”, are the starting point of fragment generation with fixed heavy atom numbers. The SMILES string is then modified to construct aliphatic rings, which are subsequently submitted for structure transformations (aromatic ring formation, sidechain rearrangement, and atom/bond replacement, etc.). A series of filters (the same filter rules in SECSE) are applied to remove fragments with undesired architecture/topology or functional groups. Final structures of 121,860,917 fragments are stored in an SQLite database (See Data availability section).

### Input preparation

In the workflow, chemical structures and protein structures are the primary inputs. Depending on different purposes, the chemical input can be an atom, a fragment structure, or a fragment library in the format of a tab-separated file containing structure SMILES and ID. If needed, the provided SMILES can be converted into a 3D structure using ETKDG v2 built in RDKit [37]. Tautomer and spiro centers are also enumerated on demand. For AutoDock Vina docking, the ligands are converted from SDF format to PDBQT format using Open Babel v3.1.1 [38]. Fragment libraries are recommended for hypothesis-driven hit discovery, especially when limited binders against the target of interest are reported. Protein 3D structures are prepared from crystal structures from the Protein Data Bank (PDB) [39]. Homology models or predicted structures from AlphaFold2 [40] /RoseTTAFold [41] are also acceptable although with compromised accuracy and predicting power. In our demo case, protein structures are prepared for docking with ADFR v1.2 [42, 43].

### Molecular generator

**Fig. 2:**
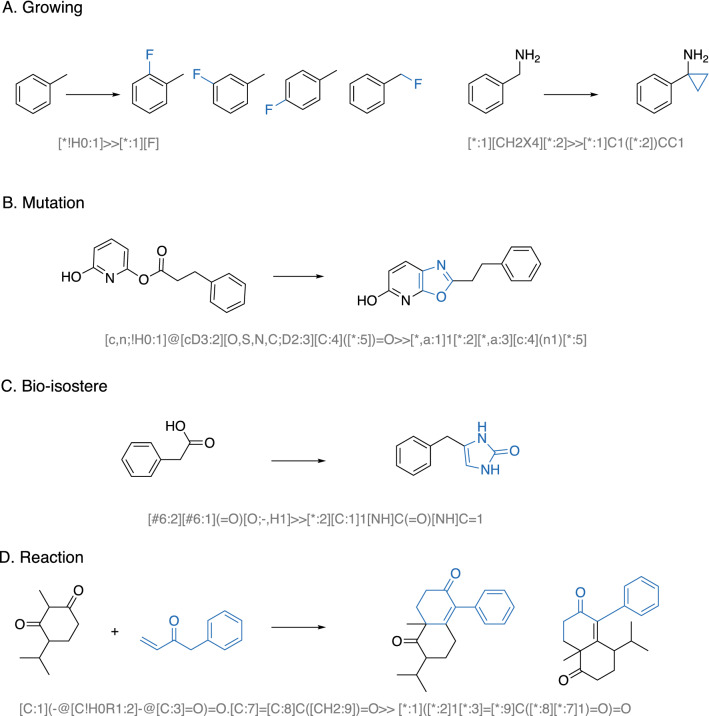
Categories of rules in SECSE and illustrative examples. **A**The Growing rule means applicable hydrogen can be replaced by a defined functional group. **B** The Mutation rule contains a large set of structural transformations commonly practiced by medicinal chemists such as ring-closing-ring-opening, insertion or deletion of atoms, and etc. **C** The Bio-isostere rule allows the interconversion of isosteric groups/atoms. **D** The Reaction rule identifies functional groups/moieties that can react with building blocks (BB) from a predefined BB library and hypothetically generates all possible products. Changed atoms are highlighted in dark blue color

The molecular generator we have developed provides a rule-based generation approach. There are four types of transformation rules (growing, mutation, bioisostere, and reaction) in our database. Some representative cases of each class are shown in Fig. 2.

1. In the grow rules, any of the replaceable hydrogen atoms on the seed compound can be replaced with a new substructure, such as an atom, a functional group, a ring, or a ring with a linking spacer.
2. The mutation rules include the following three categories: atom replacing, insertion, and deletion; ring-closing, ring-open, ring modification (expansion, reduction, contraction); as well as aromatic-aliphatic exchange.
3. The bioisostere rules refer to classical or non-classical bioisosteric replacements, which are commonly used by medicinal chemists.
4. The reaction-based rules contain common organic reactions confined to one or two steps. A library of commercial building blocks is used as starting materials. Applying the chemical reaction rule is beneficial to efficiently increase the scaffold diversity of the resulting molecules, although they can be generated from multiple rounds of rules from the previous three categories.

All the rules are represented in the reaction-like format using the SMARTS. Hybridization extension defined in RDKit was also used in the rules. A few examples from each category are provided in the SQLite database.

### Property and structure filter

To ensure that the platform generates molecules with decent chemical beauty [44], we construct several filters that define molecular properties, ring system count, and substructures.

1. The default parameters of the molecular property filters are shown as follows: molecular weight (MW) $$\in [81, 450]$$; LogP $$\in [0, 5]$$; the number of hydrogen bond donors (HBD) $$\le 5$$; the number of hydrogen bond acceptor (HBA) $$\le 10$$; the number of rotatable bonds (RB) $$\le 4$$; and topological polar surface area (TPSA)$$\le 200$$. All properties here are calculated by RDKit v2021.03.5. The bond between two connecting parts that both have π/p orbitals is not rotatable because it tends to form a conjugated system with up to two stable rotamers. To count the real rotatable bonds, the definition of RB was replaced as ’[C⌃3!D1;!$(C(F)(F)F)]-!@[!Br!F!Cl!I!H3&!$(*#*)!D1;!$([!Br!F!Cl!I](F)(F)F)]’ here.
2. The default constraints for ring systems are: total ring system count $$\le 4$$; the number of rings in a polycyclic ring system $$\le 3$$; ring size $$\le 7$$; fused ring count $$\le 3$$; bridged ring count $$\le 1$$; and spiro ring count $$\le 1$$.
3. Undesirable structures are also discarded by identity filters (sulfur, phosphorus, or structure alert), and count filters (e.g., max number of carboxylic acid or alkyne in one compound). PAINS filters are also included.

One thing worthy of note is that the filters are arbitrarily set depending on project requirements, which can be adjusted if the output is not ideal.

### Fitness evaluator

Structure-based virtual screening engines such as molecular docking or pharmacophore-based screening methods are optional for fitness evaluation. Docking is the first choice for fitness evaluation. The default docking software in our platform is AutoDock Vina v1.2.0 [45, 46]. We also provide a Glide interface for users with commercial licenses. Additionally, we offer shape-based screening and similarity scoring functions to evaluate fitness for ligand-based drug design (i.e., the initial input is not a protein structure but one or more ligands with known activity).

Several scoring functions are optimized to achieve the evaluator function for different scenarios. If the docking mode is selected, both docking score and ligand efficiency (LE) [47, 48] are considered as ranking criteria, where

$$\begin{aligned} LE = \frac{Docking\; score}{1+\ln {(Number\; of\; heavy\; atoms)}}\quad . \end{aligned}$$

The docking score tends to favor larger molecules in our previous tests. In contrast, LE can correct the issue by preventing premature enrichment of large molecules before reaching the upper molecular weight cutoff. Root Mean Square Deviation (RMSD) of aligned atoms between docking poses of the previous and current generation is calculated to determine whether the binding mode has changed in the two consecutive generations. If the similarity search mode is selected, the optional scoring functions will be a Tanimoto index of different molecular fingerprints from the generated molecules and reference compounds. In addition, the retrosynthesis module from Chemical.AI [49] is invoked to assess the synthetic availability.

### Seed selector

After scoring, molecules with RMSD less than 2 Å or with significantly decreased docking scores are selected as seeds for the next generation. The purpose of the selector is to make sure compounds with consistent binding modes are maintained while compounds with much better binding modes won’t be carved out. Then we apply a genetic algorithm [50–52] to select seeds from all eligible molecules. In our platform, the default GA operator is the tournament selection which is the most widely used selection strategy. Consequently, it can quickly converge to the optimal solution within noisy environments and introduce some randomness to avoid the limitations caused by local optimization.

Because of the limited computing resources, we sample data from the molecules generated by all the rules. Likewise, we use a partition clustering algorithm (see Additional file 1) before sampling to ensure the diversity of the selected molecules. We calculate the molecular fingerprint and Tanimoto index to evaluate the distance/dissimilarity between generated molecules, based on which the sampling is executed.

### Deep learning-based fitness prediction

Although SECSE can generate a significant number of molecules, most of them are not evaluated due to limitations in computing and storage resources. Therefore, we apply deep learning (DL) modeling to reduce computational costs and make it possible to evaluate the fitness of all molecules. We use the data generated after each generation to train the model and then predicts the fitness of unsampled molecules. Docking score or ligand efficiency can be considered as target for prediction if the docking mode was selected. Fitness prediction models are constructed using package Chemprop (version 1.3.1). Chemprop builds a directed message passing neural network and learns to predict molecular properties directly from the graph representation of molecules. [53] Two strategies are provided here for the integration of DL technology. One is the combined mode, where top-ranked molecules prioritized by predicted scores were evaluated by the fitness module. These molecules were applied for seed selection together with docked molecules from sampling procedure. Moreover, in the combined mode deep learning models will be updated with each round of molecular generation. The other one is called clean mode. The DL model is trained based on the docking results after a SECSE campaign is finished. Data from each generation can be trained independently or together. The model can then be applied on undocked molecules for fitness prediction. Molecules with good performance from DL models can be subjected for further inspection. Additionally, these two modes can be used alone or in combination.

The platform uses some open-source packages: RDKit v2021.03.5, Open Babel v3.1.1, AutoDock Vina v1.2.0, Chemprop v1.3.1, and GNU parallel v20190922 [54].

## Results

### Properties of generated molecules

We constructed a random library using SECSE without any other evaluation constraint to estimate the molecular properties of generated compounds. Benzene was assigned as the only input fragment. During each iteration, one hundred molecules that passed the filter were randomly selected as seeds for the next iteration. The final random library after ten rounds of iteration contains 2,042,863 molecules, which are included in the Figshare. More information can be found in the Data availability section.

**Fig. 3:**
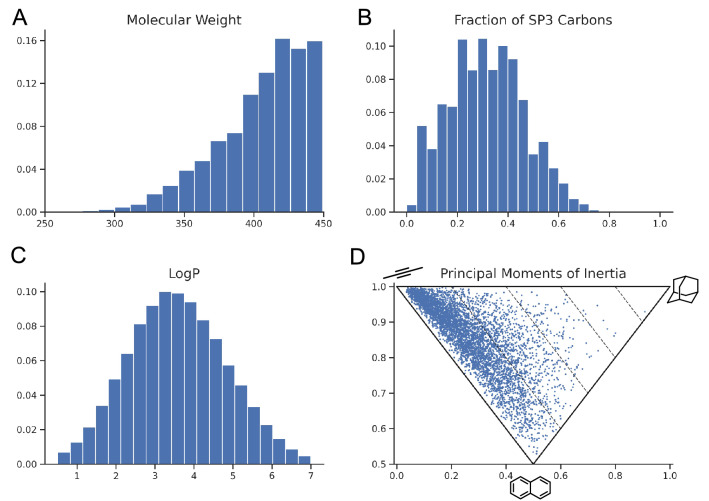
Properties of the randomly generated library. **A**–**C** shows the distribution of MW, Fsp3, and LogP of molecules in the random library, respectively. **D** The PMI plot illustrates the shape of sampled molecules from the randomly generated library. The top-left vertex represents rod-shaped, the top-right vertex represents sphere-shaped, and the bottom vertex represents disc-shaped

We calculated the physicochemical properties of the random library, such as molecular weight (MW), LogP, and the fraction of *sp3* hybridized carbons (Fsp3) [55], as well-illustrated in Fig. 3. Despite the upper limitation of MW in the filters, we could find that the peak falls around 450 Da. The distribution of LogP showed that the majority of molecules have a value between 0 and 5. Molecules with a high Fsp3 tended to be more three-dimensional in shape. The Fsp3 of the random library was well-distributed from 0 to 0.8. In addition, five thousand molecules were randomly sampled to plot the principal moments of inertia (PMI) [56], a more direct descriptor for assessing the distribution of molecular geometry (rod-shaped, disc-shaped, and sphere-shaped). We used the MMFF94 force field in RDKit to optimize the conformers of sampled molecules. As presented in Fig. 3D, the molecules scattered more towards the top-right vertex (sphere-shaped) in comparison with traditional HTS compounds that predominately dropped between the top-left vertex (rod-shaped) and bottom vertex (disc-shaped) (data not shown). HTS Compounds are usually built by two or three building blocks linearly. However, the transformation rules here can be used to extend fragments in any direction without human bias. That is why structures in our data set are more spherical than structures from other libraries designed by medicinal chemists. The results presented here indicate that SECSE can generate structures with suitable druglike properties and diverse geometry.

### Case: phosphoglycerate dehydrogenase (PHGDH)

PHGDH is a crucial enzyme that catalyzes the first committed step of the de novo serine synthesis pathway. It converts 3-phosphoglycerate to 3-phosphopyruvate in a reduced nicotinamide adenine dinucleotide (NADH)/nicotinamide adenine dinucleotide (NAD+)-dependent oxidation reaction. Many reports [57, 58] have indicated that overexpression of PHGDH is associated with various diseases, especially cancer. Inhibition of PHGDH may be a promising strategy for cancer therapy [59–63].

#### Regeneration of PDB ligands

**Fig. 4:**
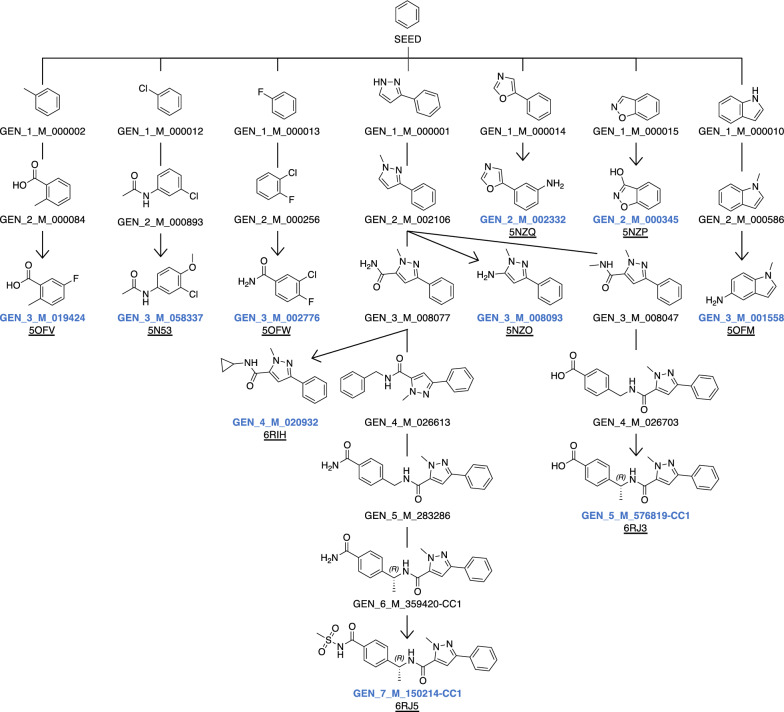
Evolution tree leading to the ligands in the co-crystal structures of PHGDH. Benzene is the initial seed. Each generated intermediate is labeled with name in black. Each reproduced structure is labeled with name in blue and an underlined PDB code

**Fig. 5:**
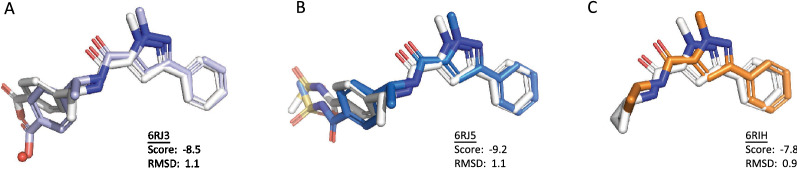
Alignment of ligands in the co-crystal structures and the corresponding reproduced molecules. The ligands from PDB 6RJ3 (A), 6RJ5 (B), 6RIH (C) are shown in white stick. The generated structures are shown in stick colored by light blue (**A**), marine (**B**), orange (**C**), respectively. The water (**A**) in the co-crystal structure is represented in red sphere. The unit of Vina score is kcal/mol. The unit of RMSD is Å

To validate the retrospective accuracy of SECSE, we performed a test to reproduce experimental binding modes for co-crystal ligands against PHGDH. By the time of our analysis, there are fourteen co-crystal structures of PHGDH containing drug-like ligands in PDB. Seven of the ligands are fragments with molecular weights of less than 150 Da. To narrow down the chemical space under limited computational resources, we selected a small set of rules (see Additional file 4) to generate molecules for this test. The set of rules can be found in the Additional file 4 in SMARTS format. The protein structure of 6RJ3 was selected as input. All water were removed from the original crystal structure. Benzene was employed as the initial fragment. AutoDock Vina was selected for molecular docking. After seven rounds of iterations, ten ligands were reproduced. The growing paths are shown in Fig. 4. Four of them evolved from the same branch and shared a common ancestor GEN_2_M_002106. Fig. 5 shows the poses and RMSD of rediscovered ligands compared to crystal conformation (6RJ3, 6RJ5, and 6RIH), respectively.

The three RMSD values are all less than 2.0 Å. Compared with the ligand in 6RJ3, the left carboxyl and phenyl of GEN_5_M_576819-CC1 are shifted, resulting in a RMSD of 1.1 Å. The reason for less constrained conformation in this part is due to removal of the crystal water at this position (shown in Fig. 5A). In addition, the other four crystallographic ligands (ligands from 6RJ2, 6PLF, 6PLG, 6RJ6) were not regenerated. The ligand from 6RJ2 was not reproduced because of the low ligand efficiency ranking of its parent molecule. In addition, ligands from 6PLF, 6PLG and 6RJ6 were not regenerated since their parent molecules did not continue to evolve as a result of insufficient sampling. The raw data can be found in Figshare (see Data Availability section).

From the test run, SECSE has proven to be powerful in the rediscovery of known ligands against PHGDH. Next, we sought to demonstrate its value in the generation of novel compounds, the transferable process of which can be applied in hit identification against novel targets with limited ligand-bound information available.

#### Generation of novel compounds

**Fig. 6:**
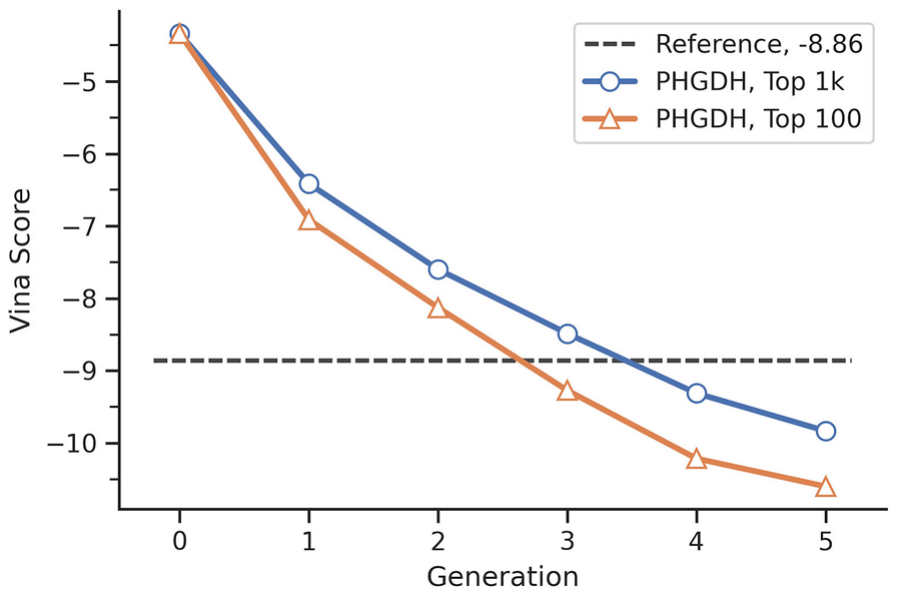
Average docking scores of top N compounds in each generation. The unit of vina score is kcal/mol. The compound in generation 0 is the initial fragment. The vina score of reference compound is -8.86 kcal/mol. The blue line represents the average vina score of top 1000 compounds. The orange line represents the average vina score of top 100 compounds

The upper limitation of molecular weight was set to 500 Da, and the starting fragment was benzene. The crystal structure of PHGDH (PDB code: 6RJ3) was prepared using previous descriptions in Implementation. Together, 502,226 poses were collected after five generations. The docking scores were gradually decreased (Fig. 6). Not surprisingly, compared with the average docking score of the top 1000 of each generation, that of the top 100 molecules was improved more rapidly. After three generations, the average docking scores of either the top 100 or top 1000 compounds were better than that of the reference compound (− 8.8 kcal/mol). Additionally, it was observed that the scores started to converge at later generations indicating the pocket occupancy was quickly approaching its optimum. It is plausible that the converging rate for different targets might be different. More rounds of iteration can be performed. Yet in this case, we stopped here for further analysis.

Finally, 14,413 poses with AutoDock Vina score less than − 9 kcal/mol were obtained. Then, the similarity distance cutoff was set to 0.15 to cluster these molecules according to the RDKit fingerprint. The one with the lowest docking score of each cluster was chosen for further binding pose inspection. Afterwards, we retrieved analogs of molecules of interest from the original docking pose pool. To keep the long-range electrostatic (LRE) interactions in the phosphate channel [62], the generated molecules with electron-rich functional groups are preferably selected using substructure filters. The final list of compounds is compiled manually. Table 1 (see Additional file 2) below includes some selected examples.

**Table 1:**
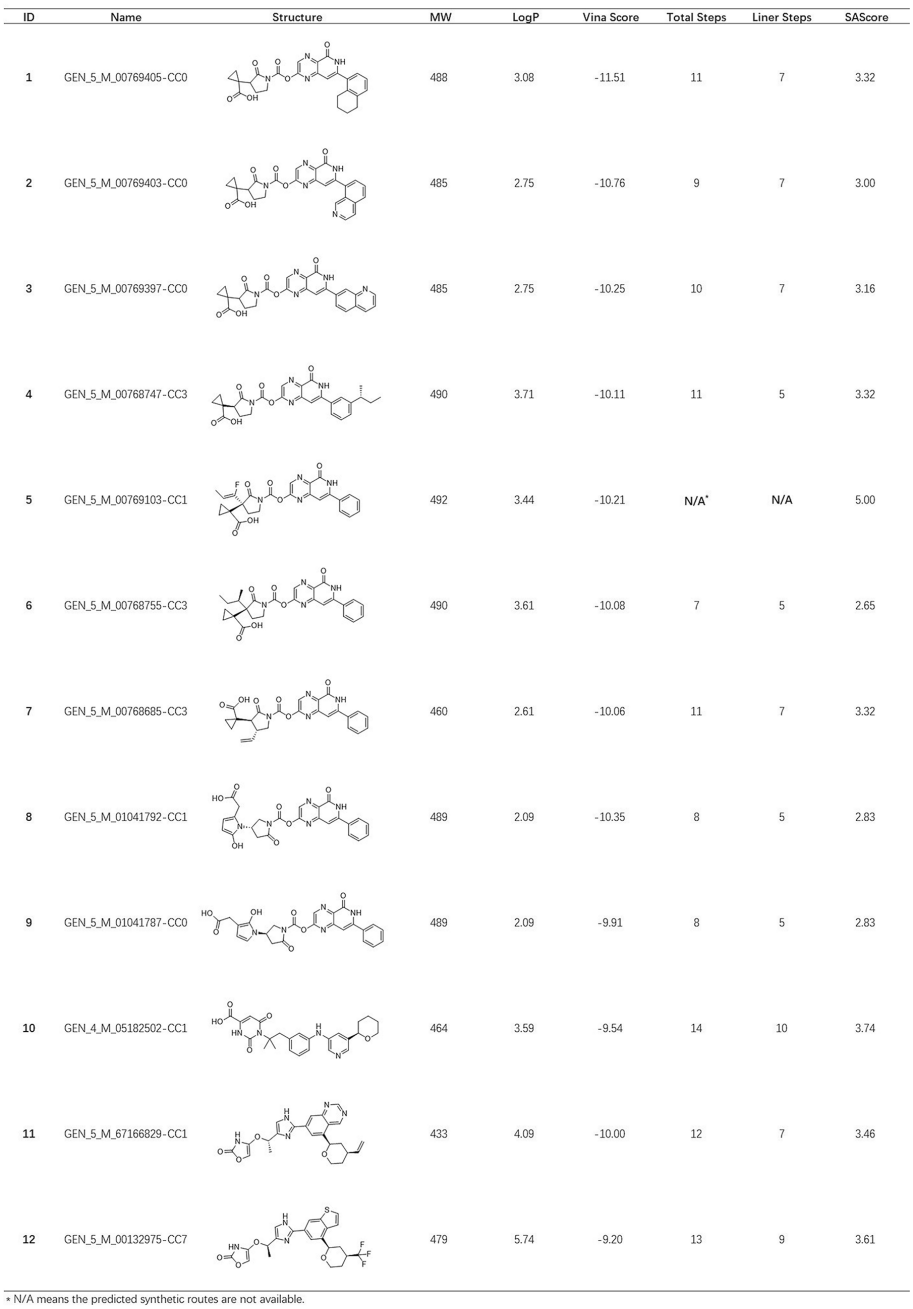
List of selected candidates

All the molecules shared similar binding modes with the reference compound from 6RJ3. Compounds **1–7** share a common topology. Similar to the phenylpyrazole-5-carboxamide part of the reference compound, the phenylpyridopyrazin-5-one occupies the same position of the adenine pocket. The nitrogen atom in the pyrazine ring forms a polar interaction with the side chain of D174 to stabilize the lipophilic aromatic fragment. The cyclopropanecarboxylic acid motif, which mimics the benzoic acid in the reference compound, has long-range Van der Waals interactions with the basic residues in the phosphate channel. The hydroxy-pyrrol-2-yl acetic acid moiety of compounds **8** and **9** act as the same role for Van der Waals interactions, as well as oxazolone of compounds **11** and **12**.

**Fig. 7:**
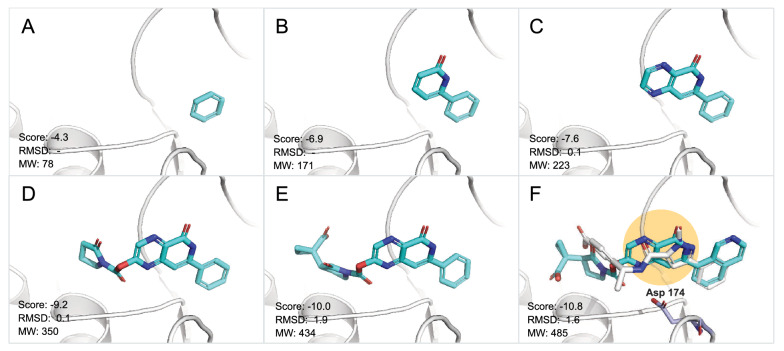
Evolutionary path of compound 2. (**A**–**E**) fragment binding poses of Compound **2** in generation 1-5. **F** binding pose of compound **2** and reference compound in the NADH/NAD+ binding pocket. The protein structure is shown in white cartoon. Compound **2** is shown in cyan stick. The reference compound is represented as white stick. Asp 174 is shown as lightblue stick. The adenine pocket is highlighted in a yellow circle. Vina score, RMSD(Å) of shared structure between two consecutive generations, and MW(Da) are provided

The step-by-step elaboration of compound **2** from the benzene ring in the NADH/NAD+ binding pocket was presented in Fig. 7A to F. The final pose of compound **2** was aligned to the reference compound. The common structure between current and previous generations showed nearly identical orientations. The AutoDock Vina docking scores decreased from − 4.3 kcal/mol to − 10.8 kcal/mol, while the MW increased from 78 Da to 485 Da. Despite the decent docking scores, conformation of compounds in D, E, F generated by AutoDock Vina may not be energetically favorable. The oxygen atom of the carbonyl group is near the nitrogen atom of pyrazine, whereas they should stay away in the lowest energy conformations. More accurate docking programs are needed for better outcomes.

Subsequently, the Synthetic Accessibility Module from Chemical.AI was used to estimate accessibility of these proposed molecules. The Synthetic Accessibility Module provides a primary estimation for many organic compounds under restricted computing resources. The predicted routes may not be the best choice, but it gives a quick estimate that can be used to assess whether the compound is easy to make or not. Generally speaking, majority of compounds can be made within 15 synthetic steps with no more than 7 linear steps. Unfortunately, no synthetic routes of compound **5** are suggested under the default setting of Synthetic Accessibility Module. In such cases, or when dealing with a short list of candidates that are of high interest, more accurate predictions can be done using the Synthesis Plan Module, which performs extensive searches for all possible synthetic routes.

**Fig. 8:**
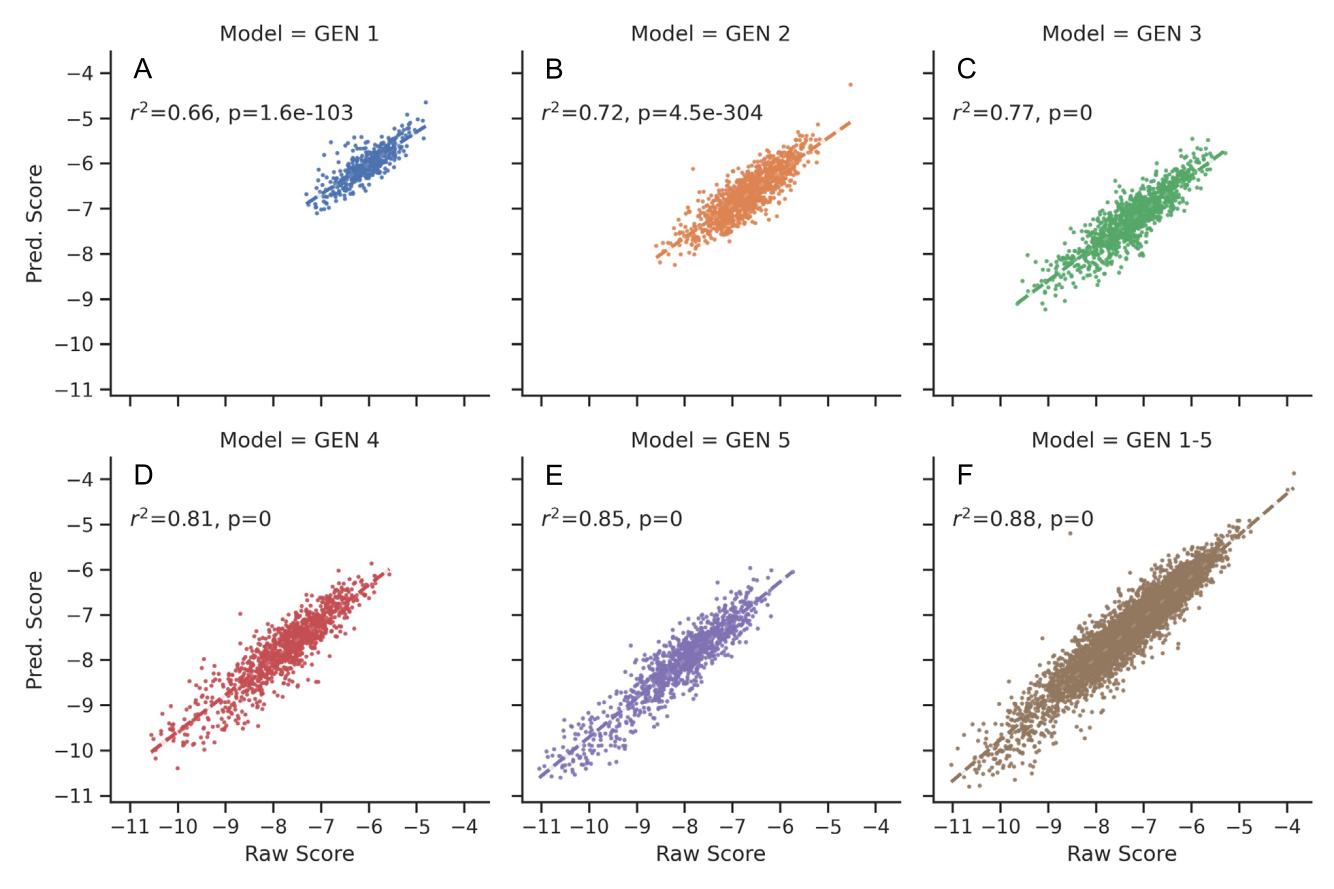
Test data $R^{2}$ of deep learning models. All the docking data are randomly split into three parts: training (80%), test (10%), and validation (10%) datasets. **A**–**E**) shows the performance of models using docking data set from generation 1–5, respectively. **F** shows the performance of the aggregate dataset, including data from the previous five generations

To address the sampling limitation of generated molecules before fitness evaluation, a new selection protocol combined deep learning method was developed. As described previously in the Implementation section, the clean mode was used to build the DL model. Fig. 8 demonstrates the details of the model performance of each generation (A-E) and combined set (F), which includes data from all previous generations. The value of $R^{2}$ from Generation 1 to Generation 5 was gradually improved from 0.66 to 0.85. Furthermore, the $R^{2}$ of the DL model from the combined set was 0.85, slightly better than other models trained only by a single generation. It is reasonable to believe the model performance is sufficient for prediction [64–66]. The model F was then used for the prediction of the 66,687,173 molecules and 2,094 molecules with predicted scores less than − 10.5 kcal/mol were subjected for redocking. The structures, MW, LogP, and synthetic accessibility analysis of representative molecules were listed in Table 2 (see Additional file 3). It was pleased to see compounds that share similar scaffold with compound **2**. Compounds that have completely different scaffolds were also identified to provide diverse and valuable hypotheses for further validation. The superior performance of the deep learning model in the SECSE platform was speculated to result from the intrinsic logic. The 3D structural information of the parent compound was inherited in its child compounds, while the child generation would feedback rich structure-activity relationship (SAR) for model training. It also explained the model performance was improved in later generations while the combined set yielded the best result.

**Table 2:**
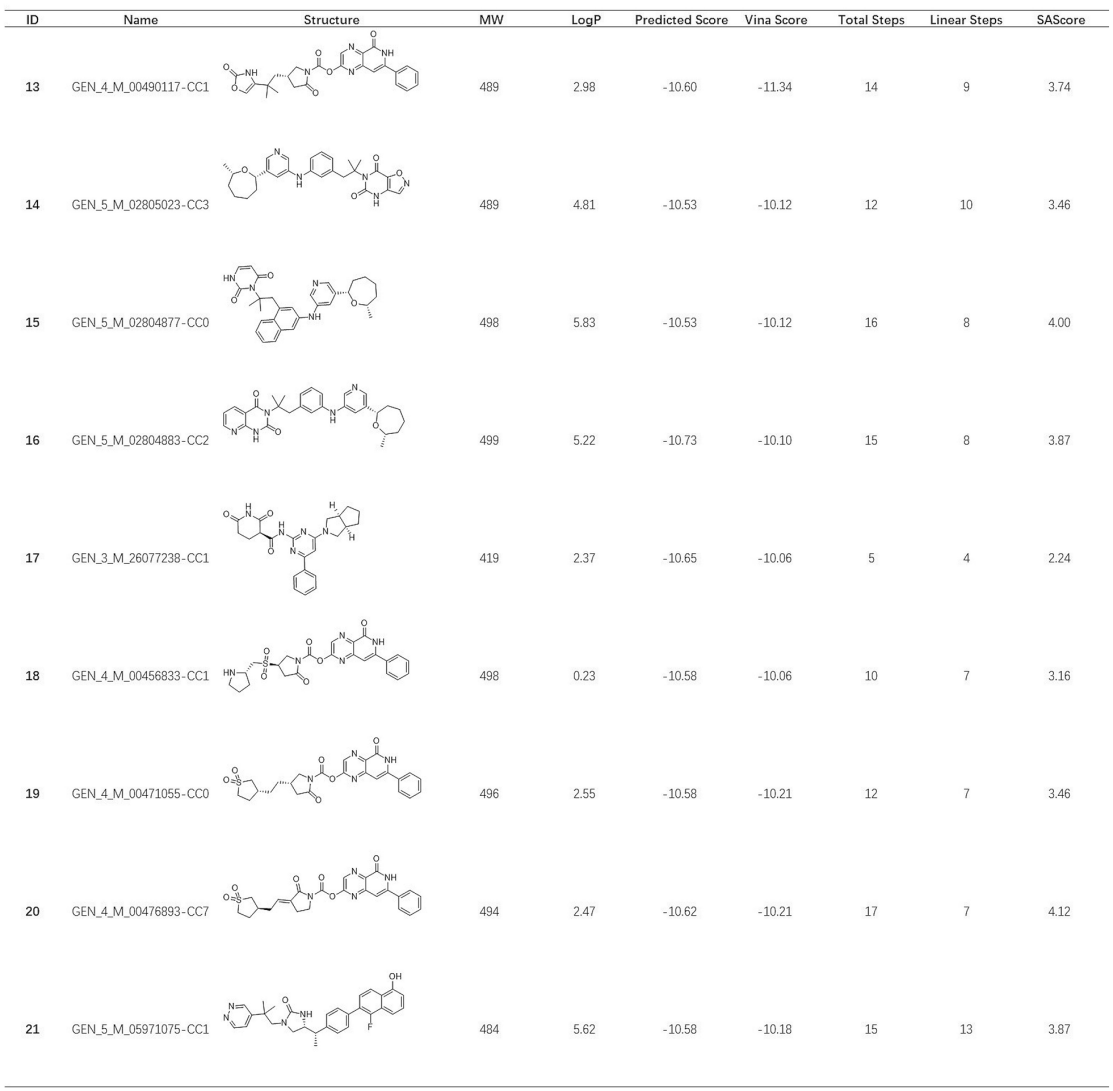
List of selected candidates based on the evaluation of DL models

**Fig. 9:**
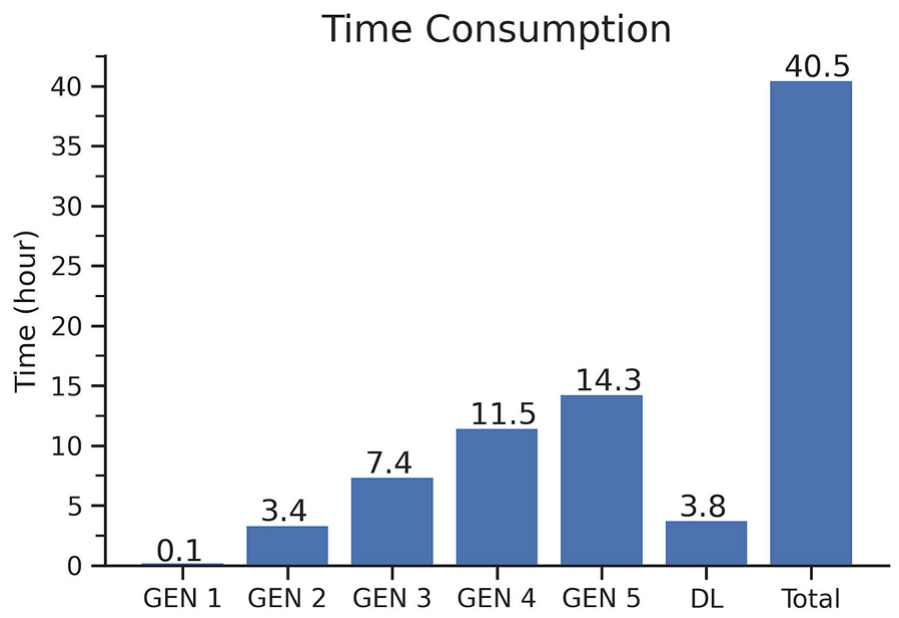
Time consumption. Running time of each generation (GEN 1–5). DL represents the time cost of a final search by deep learning model. With 80 cores, a five-generation computing cost was 40.5 hours

In this case, we used an 80-core computer, which took 40.5 hours in total. The Fig. 9 shows the calculation time of each generation. As the molecules grow larger and their complexity increases, the running time of each generation would gradually increase. Deep learning modeling significantly reduces search time and makes it possible to obtain estimated fitness scores for all generated molecules.

## Discussion

To our knowledge, the previously reported de novo design tools are not widely used in hit-finding programs. A potential reason is the inadequate exploration of chemical space. Moreover, limitation in chemical space coverage of current chemical libraries is a common problem the field is facing. Intensively expanding the chemical space via brute-force exploration, which leads to ultra-large chemical libraries such as GDB [36, 67, 68] is explored. A more widespread attempt is the make-on-demand library [4], which comprises of structures from the enumeration of commercial building blocks based on reliable reaction schemes. The commercial providers also claim to have a relatively high synthesis success rate (at least 30%). The success of ultra-large compounds virtual screening contributes to the vigor of the make-on-demand library [2, 3]. Furthermore, people train machine learning models to accelerate the speed of virtual screening to balance the tradeoff between accuracy and speed [64]. However, it is still a very tough task to do virtual screening of ultra-large libraries directly on the present hardware.

All these factors are considered and balanced in our own platform. It is probably unrealistic to enumerate all druglike molecules, but an exhaustive enumeration of fragments with less than 13 heavy atoms is doable. Compared with previous de novo design softwares, reaction rules are a systemically curated to enrich the diversity of structures. Many heulistic filters from medicinal chemists’ knowledge were incorporated in SECSE to maintain the beauty and practicality of molecules. To avoid the combinatorial explosion, protein pockets are constraints to direct the evolution. In addition, the unexpected accuracy of the deep learning model allows us to evaluate a large amount of compounds with minimal false positives or false negatives.

Recently, deep generative neural networks have become a promising approach for molecular generation. Many seminal reviews [10, 69] have summarized the development of these deep generative models with different generative architectures (like recurrent neural networks, autoencoders, and generative adversarial networks) based on various molecular representations (SMILES, molecular graph). Despite the limitations of these generative models and the inaccuracy of current evaluation techniques for these models [70], they are indeed one choice for de novo molecular generation.

In parallel, rule-based molecular generation is also very popular such as AbbVie’s project Drug Guru [34, 35], the abbreviation of drug generation using rules. A data-driven method called matched molecular pairs (MMPs) [71–73] is another way to collect the experts’ knowledge from literature. Indeed, the rules of Drug Guru and MMPs are essentially the same method and nearly from the same source, that is molecular design thoughts of human beings. They can be stored as lines of reaction SMARTS code in RDKit. Scientists from Shanghai Institute of Materia Medica constructed DrugSpaceX [74], a virtual compound library, using Nova and BIOSTER rules from StarDrop. Scientists from GSK did a Turing test [75, 76] for molecular generators by comparing three molecular generators in-house. The first one is BioDig, an MMPs-based algorithm. The second one is BRICS, a molecular generator by fragment recombination. The last one is RG2Smi, a deep generative model for generating molecules, which translates a molecule into a pharmacophore-based graph representation, then generates smiles string by deconvolution algorithm trained using natural language processing architecture. BioDig performed better than the other two methods across all tests in their report. Despite the fact that rule-based methods are somewhat limited to human knowledge or bias, we prefer rule-based methods for practical considerations.

Another challenge in computational de novo drug design is that compounds proposed by these tools are often hard to synthesize. Therefore, synthetic accessibility is a critical assessment for meaningful output. Previous retrosynthesis analysis tools were usually incapable of handling complex synthetic routes. Recently, with the development of deep learning and the growing number of available reaction collections, several new algorithms [77–79] have been developed to improve the capability of synthetic route planning. We introduce synthetic accessibility evaluator of Chemical.AI to analyze the feasibility of generated structures. Yet increasing the evaluation throughput is challenging since it may take a few minutes to find practical routes for a single molecule. A batch mode that can evaluate thousands to millions of molecules at an affordable cost within a given timeframe is urgently needed.

SECSE mainly relies on structure-based computational design tools. Different tools will lead to different search directions, which might result in different chemical structure output. SECSE platforms are built to be compatible with various tools as fitness evaluators, like molecular docking, shape-based screening, and pharmacophore alignment, even ligand-based screening methods. Until now, docking has been the primary choice of SECSE because of its tradeoff between accuracy and speed. Despite the excellent performance of SECSE docking mode, there are still some inherent shortcomings in molecular docking methods, such as simplistic scoring with empirical energy function, rigid protein structures, ill-modeled poses. To enhance the prediction power, theoretically more accurate methods need to be introduced into the fitness assessment module. Claudio *et al.* [80] proposed a new QM-based docking program to replace the current docking methods based on molecular mechanics (MM) force fields. The new scoring function has achieved excellent performance in most cases. However, their QM docking scoring function is ten times slower than traditional MM-based scoring functional per core. To explicitly consider the dynamic nature of proteins, molecular dynamics (MD) simulation is the best choice. Hugo Guterres *et al.* [81] reported a high-throughput molecular dynamics (HTMD) simulations method to refine the docking results from AutoDock Vina. They calculated the RMSD of ligand by aligning protein structure from the initial docking pose and all protein structures in MD trajectory. They used a large set of 56 diverse target proteins and 560 ligands from the DUD-E dataset. The results show that short time MD simulations increase the area under the curve (AUC) of 0.8 from a value of 0.68 from AutoDock Vina. Enabled by the increasing computational power, attempts to add QM and MD concepts to the current docking program will be a promising way to improve the fitness evaluation module of SECSE. Results of the application of SECSE in our internal research projects will be reported in due course.

## Conclusion

We have developed a de novo design platform SECSE that integrates human intelligence for systemic evolutionary chemical space exploration against a specific protein pocket. The platform incorporated design rules of medicinal chemistry, computational evaluation methods, and deep learning models to efficiently speed up the search process of virtual hit compounds. The application in a demo target PHGDH proved its utility in finding diverse, potent and novel drug-like chemotypes. Further optimization considering high-precision evaluation methods and protein dynamics is currently underway. SECSE is released as an open-source project under the Apache License, Version 2.0. Any efforts and suggestions to improve its performance are welcomed.

## Supplementary Information

**Additional file 1: **Fragment library generation and clustering algorithms.

**Additional file 2: Table S1.**SMILES and releated information of molecules from Table 1.

**Additional file 3: Table S2.** SMILES and releated information of molecules from Table 2.

**Additional file 4:** Rules for regeneration test in SMARTS format.

## Data Availability

Fragment Library: 10.6084/m9.figshare.17142236. Random Library: 10.6084/m9.figshare.19142624. Raw data of regeneration test : 10.6084/m9.figshare.19235217. Raw data of demo case PHGDH: 10.6084/m9.figshare.17141879.

## Abbreviations

HTS: High-throughput screening
AS-MS: Affinity selection-mass spectrometry
FBDD: Fragment-based drug design
DELT: DNA-encoded library technology
VS: Virtual screening
MW: Molecular weight
HBD: Number of hydrogen bond donors
HBA: Number of hydrogen bond acceptor
RB: Number of rotatable bonds
TPSA: Topological polar surface area
RMSD: Root Mean Square Deviation
DL: Deep learning
PDB: Protein Data Bank
Fsp3: Fraction of *sp3* hybridized carbons
PMI: Principal moments of inertia
PHGDH: Phosphoglycerate Dehydrogenase
NADH: Reduced nicotinamide adenine dinucleotide
NAD+: Oxidized nicotinamide adenine dinucleotide
LRE: Long-range electrostatic
MMP: Matched molecular pair
MM: Molecular mechanics
MD: Molecular dynamics
QM: Quantum mechanics
HTMD: High-throughput molecular dynamics
AUC: Area under curve
SAR: Structure-activity relationship
SA: Synthetic accessibility
