# Supplementary material for: Systemic evolutionary chemical space exploration for drug discovery
Source: J Cheminform. 2022 Apr 1;14:19. doi: 10.1186/s13321-022-00598-4 (PMC8973791; doi:10.1186/s13321-022-00598-4)
Supplement: Supplementary file 1 — Additional file 1: Fragment library generation and clustering algorithms. [file 13321_2022_598_MOESM1_ESM.pdf]

Supporting Information

Systemic Evolutionary Chemical Space Exploration for Drug Discovery

Chong Lu,[†,¶] Shien Liu,[†,¶] Weihua Shi,[†] Jun Yu,[†] Xiaoxiao Zhang,[†] Zhou Zhou,[†] Xiaoli Lu,[†] Faji Cai,[†] Ning Xia,[‡] and Yikai Wang*,[†,§]

†*Keen Therapeutics Co., Ltd.*

‡*Chemical.AI*

¶*Contribute equally.*

§*Corresponding Author*

E-mail: wang_yikai@keenthera.com

Fragment Library Generation

As is shown in Figure S1, a carbon string can be systemically closed to form rings with the same heavy-atom count. Sidechain replacement here means three connected non-aromatic atoms were rearranged to a center atom with two branches. The Enumerate Heterocycles function in RDKit is applied for heterocyclic ring generation if a ring structure exists. Reaction rules are used to achieve hetero atom replacement, bond replacement, and aromatic ring conversion. All the codes for fragment construction is available at `http://github.com/KeenThera/fragment_generation`.

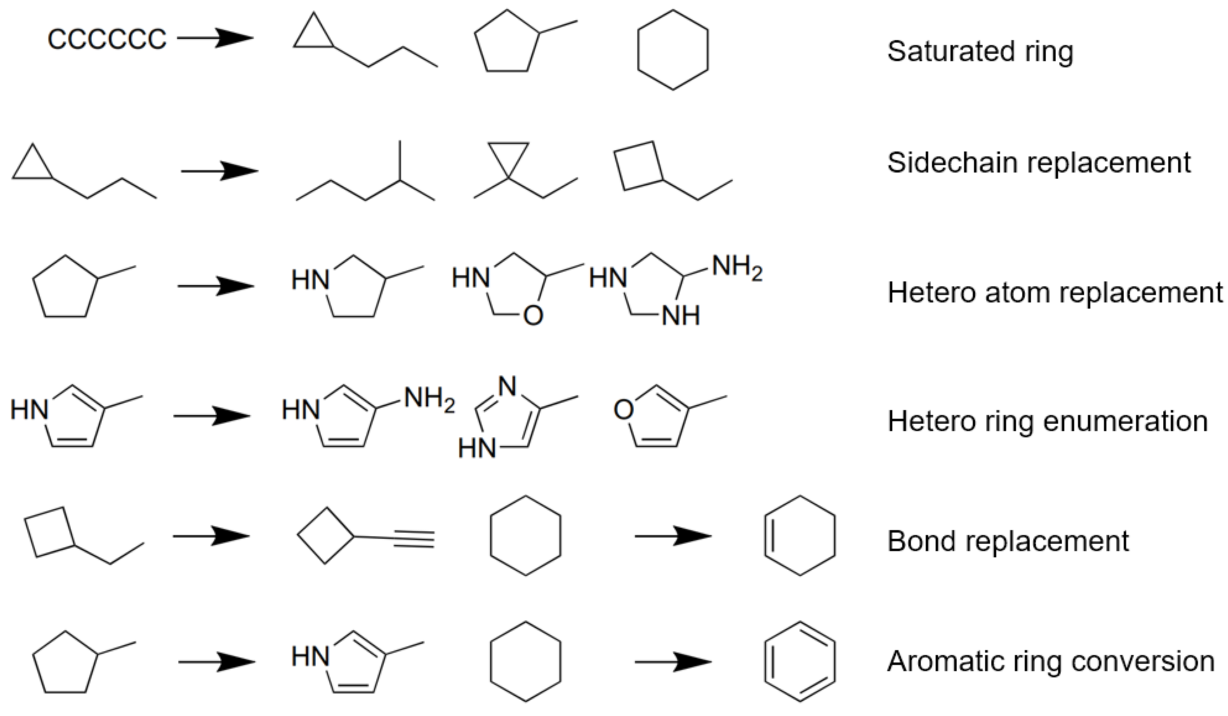

Figure S1: Examples of fragment construction rules.

Clustering Algorithms for Huge Dataset

Figure S2 presents the algorithm of clustering used in SECSE for selection of representative molecules. Clustering is necessary at this stage for efficiency considerations. In addition, a partition clustering method is introduced to reduce the computational cost significantly. Fingerprints calculated by RDKit (e.g., Morgan/Circular, MACCS keys) of all the molecules in the dataset are calculated as input features. Firstly, we randomly labeled one molecule in the dataset as the first cluster center C_1. Then, we calculated the distance/dissimilarity $(1 - Tanimoto\ index)$ of all the rest molecules with the first cluster center C_1. The molecule with the largest distance is labeled as the second cluster center C_2. At the same time, molecules that are pretty similar to the first cluster center will be masked. Next, the molecule with the largest distances to C_1 and C_2 would be considered the third cluster center C_3; molecules close to C_2 will be masked. Same iterations will continue until we find enough cluster centers or convergence is reached. Finally, we calculated the distance between all non-

cluster center molecules and cluster centers and then assigned them based on the nearest cluster center id.

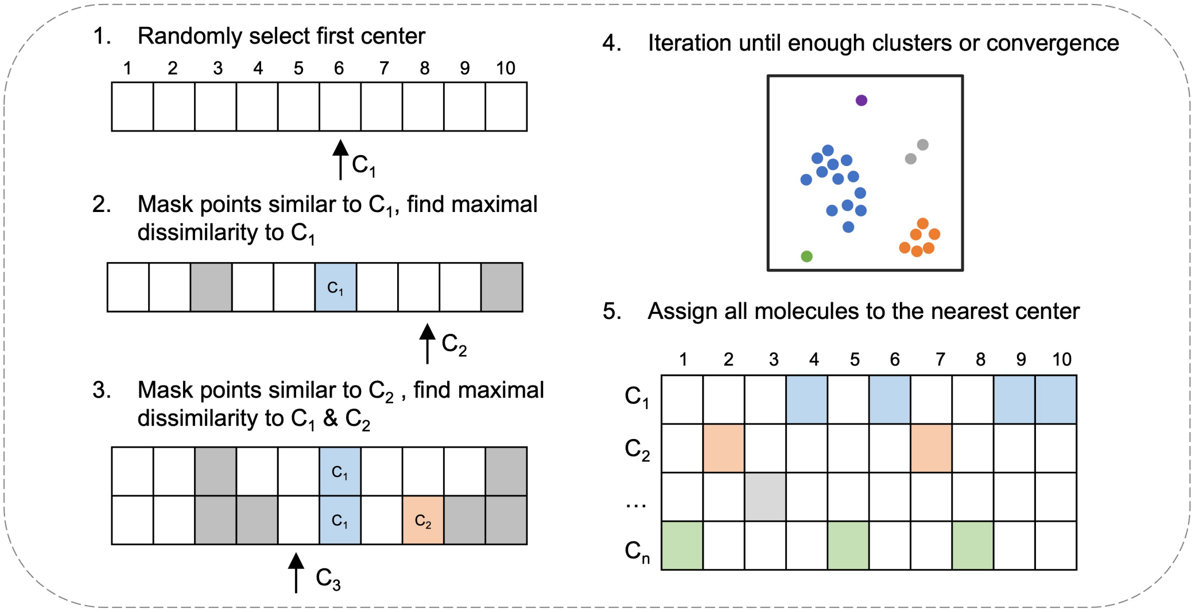

Figure S2: Details of clustering algorithms
